# Untargeted Metabolome Analyses Revealed Potential Metabolic Mechanisms of *Leymus chinensis* in Response to Simulated Animal Feeding

**DOI:** 10.3390/ijms25116110

**Published:** 2024-06-01

**Authors:** Chunxu Zhou, Ruiqi Zhao, Han Wang, Bao Liu, Yingjie Yu, Lili Jiang

**Affiliations:** 1 College of Life Sciences, Jilin Normal University, Siping 136000, China; 2 Key Laboratory of Molecular Epigenetics of MOE, Northeast Normal University, Changchun 130024, China

**Keywords:** forage, metabolome, animal feeding, metabolic pathway, flavonoid

## Abstract

*Leymus chinensis* (Trin.) Tzvel., also known as the “Alkali Grass”, is a major forage grass in the eastern and northeastern steppe vegetation in the Songnen Prairie. It is of great practical significance for grassland management to understand the influence of animal saliva on *L. chinensis* during animal feeding. In this study, we used clipping and daubing animal saliva to simulate responses to grazing by *L. chinensis*, and analyzed the physiological and metabolomic changes in response to simulated animal feeding. Results showed that the effects of animal saliva on physiological and metabolic processes of the treated plants produced a recovery phenomenon. Moreover, the effects of animal saliva produced a large number of differential metabolites related to several known metabolic pathways, among which the flavonoid biosynthesis pathway has undergone significant and persistent changes. We posit that the potential metabolic mechanisms of *L. chinensis* in response to simulated animal feeding are closely related to flavonoid biosynthesis.

## 1. Introduction

*Leymus chinensis* is a perennial forage grass possessing many favored properties including high yield, good palatability, high feeding value, and salt-alkali tolerance [1]. Recent studies have shown that *L. chinensis* can adopt different strategies in response to variable degrees of grazing intensity [2,3]. For instance, it was found that its photosynthetic capacity decreases under light grazing intensity, and the damaged photosynthetic apparatus accumulates reactive oxygen species (ROS). However, the net photosynthetic rate (P_n_) can be increased, promoting regrowth and photo-assimilation. With the increase in grazing intensity, photosynthetic electron transfer was significantly reduced. However, the damage of ROS can be mitigated by increasing energy dissipation, and the plant hormone abscisic acid (ABA), Jasmonic acid (JA), and salicylic acid (SA) signals increased a sucrose transporter gene (*LcSUT1*) expression and water-use efficiency that stored carbon underground to promote survival. Under heavy grazing, the chloroplast ultrastructure can be destroyed, and the adjustment of internal mechanisms increased the compensatory photosynthesis. Meanwhile, the increased tillers can promote the regeneration after grazing. Moreover, seasonal grazing also affected the growth of *L. chinensis* [4]. The regeneration capacity was significantly improved under early summer grazing, and the proline content and antioxidant enzyme activity were higher, and the stress resistance was stronger, while it was less affected under spring grazing. In a word, both the intensity and time of grazing can affect the growth of *L. chinensis*, and suitable grazing conditions can improve its stress resistance and regeneration ability.

The coevolution between plants and herbivores dates back to 400 million years ago and constitutes a complex interplay [5]. Herbivores obtain the nutrients they need from plants, and plants take a variety of defense mechanisms to reduce the damage caused by herbivores [6]. Plants have evolved diverse self-defense mechanisms against herbivores’ attacks, including both physical and chemical barriers [7]. Physical barriers include the development of spines, trichosomes, and cuticles, while chemical barriers include secondary metabolites (SMs) and volatile organic compounds (VOCs). These chemicals both provide information in the plants being attacked, mediating the relationship between the plant and the herbivores, and send warning signals to neighboring plants [6,8]. In addition, many chemical and morphological characteristics of plants are also used as defenses against herbivores [9]. When animal feeding occurs, plants perceive the clipping and saliva by the herbivores, producing ion fluxes to change plasma membrane potential (Vm), the generation of intracellular calcium (Ca^2+^) and ROS to trigger signal transduction [7,10]. In the process, various plant hormones (such as jasmonic acid, salicylic acid, and ethylene) are produced, VOCs and SMs are released, and ultimately defense effects are achieved. It is worth mentioning that the response at plant wounds plays a central role in plant defense mechanisms [11]. In this process, the plant hormone jasmonic acid (JA) is an important substance, and it can have a rapid and brief outbreak at the plant wound, then acts as a signal mediating the production of a large number of secondary metabolites, further endowing the plant with resistance [12]. Such metabolic changes in plants have been shown to be important in plant–herbivore interaction, and the strength of the interaction is often mediated by different secondary metabolites [13,14]. There are thousands of low-molecular-weight organic compounds in plants, including primary metabolites needed for plant growth, secondary metabolites needed to mediate plant interactions with the environment, and hormones that regulate organic processes and metabolism [15]. Among them, secondary metabolites are multifunctional, and they can act as effective regulators of plant growth and defense [16]. In addition to the important role of JA, the saliva of herbivores is also very important in the process of animal feeding. For example, Li et al. compared the effects of water, sheep saliva, epidermal growth factor (EGF), thiamine, and the mixture of EGF and thiamine on the growth of clipped *L. chinensis* and found that the biomass and buds increased after sheep saliva treatment rather than water treatment, although the animal saliva components had no cumulative effect on plant growth [17]. Moreover, animal saliva can also affect the level of hormones in plants [18]. Deer saliva activated salicylic acid in beech clipped leaves and leads to an increase in cytokinin in clipped beech buds. Meanwhile, the application of deer saliva to clipped maple leaves also resulted in several hydrolyzed tannins (mainly ellagitannins) and increased biosynthesis of flavonols. All in all, the herbivores’ saliva can produce physiological, metabolic, and hormonal effects on plants. However, the metabolic mechanisms by which plants respond to animal saliva are still unknown.

The comprehensive and quantitative analysis of various metabolites in biological samples is possible thanks to the rapid technological progress in metabolomics [19]. For example, the response of *Volvariella volvacea* to low-temperature stress at different time points by metabolomic analysis was found to mainly involve amino acid metabolism, carbohydrate metabolism, the TCA cycle, energy metabolism, and other metabolic pathways [20]. Metabolome analysis can also be applied in combination with other omics. For instance, the combined analysis of metabolomic and transcriptomic data of *Liriope spicata* showed that flavonoid biosynthesis, carbohydrate metabolism, amino acid metabolism, lipid metabolism, and the signal transduction pathway were significantly enriched under freezing stress [21]. With rapid progress in the omics’ era, new methods are available for the study of plant–herbivore interaction [22]. Transcriptomic analysis of cloned *L. chinensis* showed that grazing had a transgenerational effect on its growth inhibition and led to the development of a dwarf phenotype in response to the severe degradation of the grassland habitat due to heavy grazing [23]. In addition, transcriptomic analysis of overgrazed *L. chinensis* showed that most differentially expressed genes (DEGs) were significantly enriched in phenylpropanoid biosynthesis and flavonoid biosynthesis pathways [24]. Therefore, through the analysis of omics, we can find relevant DEGs, differentially expressed metabolites (DEMs), and other information, enabling the identification of key metabolic pathways, pinpointing the crucial metabolic response mechanisms and identification of the causal genes.

In this study, the grazing process was simulated by clipping and daubing animal saliva, and the widely untargeted metabolomics analysis was used to explore differential metabolites and metabolic pathways that may be associated with the response to simulated animal feeding in *L. chinensis* at the molecular level. Our results may provide new strategies for improving the quick recovery of *L. chinensis* after animal feeding, as well as more effective grassland management.

## 2. Results

### 2.1. Accumulation of Oxidative-Stress-Related Factors in Leymus chinensis Experiencing Simulated Animal Feeding

**Figure 1:**
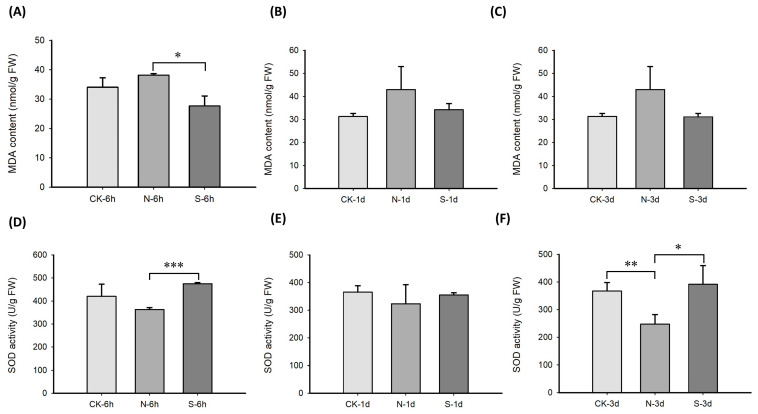
Determination and analysis of antioxidant index in leaves of *L. chinensis*. (**A**) MDA content at 6 h. (**B**) MDA content on day 1. (**C**) MDA content on day 3. (**D**) SOD activity at 6 h. (**E**) SOD activity on day 1. (**F**) SOD activity on day 3. (* *p* < 0.05, ** *p* < 0.01, *** *p* < 0.001). CK represents the control groups. N represents the clipping treated groups. S represents the animal saliva treated groups.

The malondialdehyde (MDA) content in leaves of *L. chinensis* increased 6 h after clipping, while it decreased significantly in leaves smeared with animal saliva after clipping (*p* < 0.05) (Figure 1A), indicating that the damage to cells caused by clipping was attenuated. Moreover, the MDA content smeared with animal saliva reverted to a level close to those of the control group on day 1 and 3 post the treatment (Figure 1B,C). Therefore, it appeared that animal saliva plays an important role in wound repair at the early stage. As one of the functions of plant defense system, Superoxide Dismutase (SOD) can remove superoxide anions in cells to produce H_2_O_2_. Thus, the activity of SOD was also determined in this study. SOD activity data showed that 6 h after clipping treatment, the activity of SOD began to decline, while it showed a very significant increase with animal saliva after clipping (*p* < 0.001) (Figure 1D). Moreover, this trend was also observed on day 1 after the treatment (Figure 1E). The SOD activity decreased significantly (*p* < 0.01) on day 3 after clipping; meanwhile, it showed a significant increase with animal saliva after clipping (*p* < 0.05) (Figure 1F). This suggests that when plants were under stress, intracellular free radicals accumulated rapidly, then SOD and other protective enzyme systems were destroyed, leading to a decrease in SOD activity and the accumulation of harmful peroxidation products, such as MDA. However, SOD activity in the leaves of *L. chinensis* significantly increased after application of animal saliva, which suggests the regulation of animal saliva on the cell-oxidation-related process.

### 2.2. Overall Assessment of Untargeted Metabolomic Data in Response to Simulated Animal Feeding in L. chinensis

**Figure 2:**
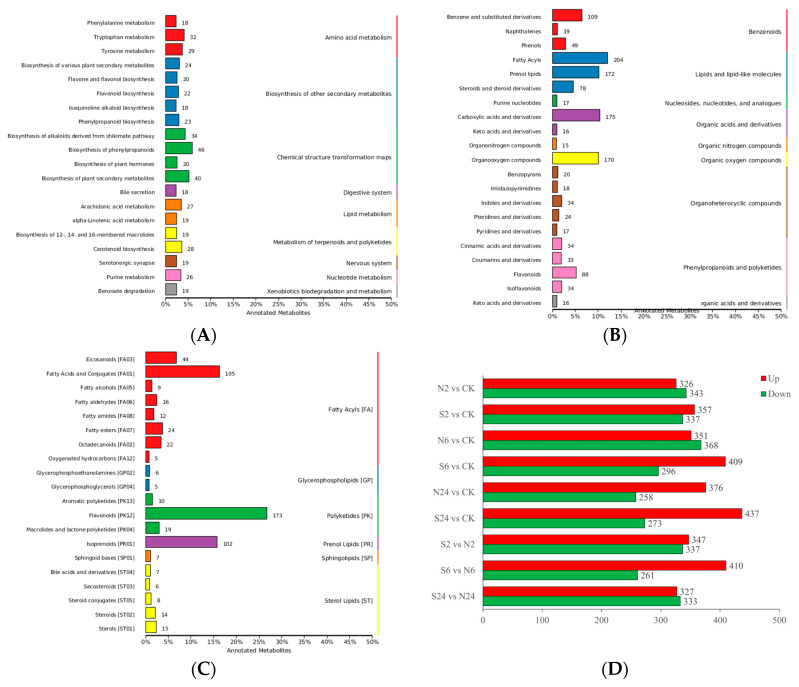
Overview of all metabolites. (**A**) The top 20 metabolites annotated in KEGG database. (**B**) The top 20 metabolites annotated in HMDB database. (**C**) The top 20 metabolites annotated in Lipidmaps database. (**D**) The number of differential metabolites between different comparison groups (Metabolite information annotated from the KEGG database).

Based on the LC-QTOF platform, a total of 25,907 peaks were detected in the default mode (positive ion mode + negative ion mode), and 4635 metabolites were identified. Among these metabolites, the number of metabolites annotated by the Kyoto Encyclopedia of Genes and Genomes (KEGG) database was 1171, accounting for about 25% of the total identified metabolites. Meanwhile, in the Human Metabolome Database (HMDB) and Lipidmaps database, 1714 and 646 metabolites were annotated, respectively. The top 20 metabolites annotated in each database are shown in Figure 2A,B, and C, respectively, and the detailed classification of each database is shown in the Supplementary Materials (Tables S1–S3). According to Fold Change ≥ 1, VIP ≥ 1, and *p*-value < 0.05, differential metabolites annotated in nine comparisons were obtained through further analysis in the KEGG database (Figure 2D).

**Figure 3:**
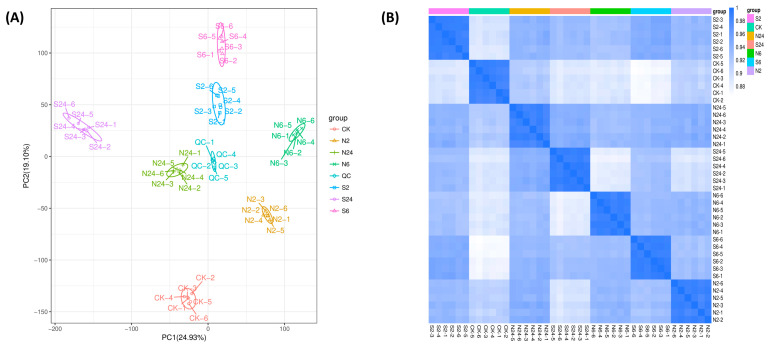
Correlation analysis of all samples. CK is the control group; S2, S6, S24, N2, N6, and N24 are the treatment groups. (**A**) Principal component analysis (PCA). QC is the quality control sample. (**B**) Samples correlation among the 42 samples. The heatmap represents Spearman’s rank correlation coefficients between each sample.

The principal component analysis (PCA) performed on all the samples (Figure 3A) showed that there were few differences within each group, and the six biological replicates in each group were all within the 95% elliptical confidence interval, while there were significant differences between groups, and the QC samples also showed obvious aggregation, which indicated that the instrument was stable and the results were reliable. The two principal components of PCA together explained 44.03% of the total variance, and the first principal component (PCA1) explained 24.93% of the total variance and clearly separated the groups. Pearson correlation coefficients were calculated for differential metabolites between pairwise comparisons of the control group (C), clipping group (N), and simulated animal feeding group (S). The closer the correlation coefficient towards 1, the greater the similarity of the samples. Results showed that the Pearson correlation coefficient is mostly close to 1 between biological replications of each treatment group, so the differential metabolites detected in this study were reliable (Figure 3B).

### 2.3. Analysis of Two Situations Resulting from the Action of Animal Saliva

**Figure 4:**
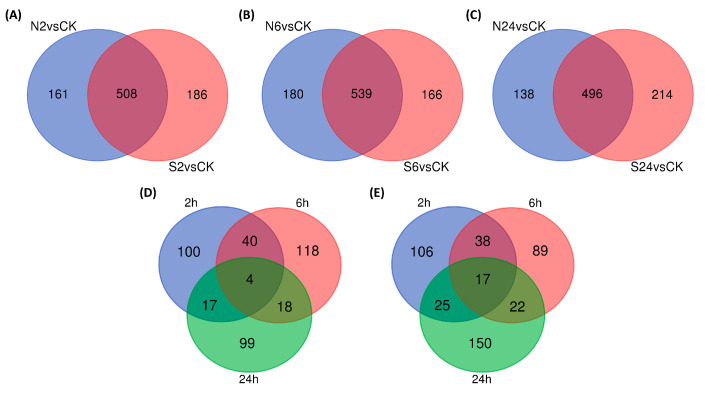
Differential metabolite analysis from the action of animal saliva. (**A**) Venn diagram of differential metabolites after 2 h of animal saliva treatment. (**B**) Venn diagram of differential metabolites after 6 h of animal saliva treatment. (**C**) Venn diagram of differential metabolites after 24 h of animal saliva treatment. (**D**) The total Venn diagram of differential metabolites in the recovery group. (**E**) The total Venn diagram of differential metabolites in the saliva-specific group.

A large number of differential metabolites were produced at 2 h, 6 h, and 24 h after the clipping treatment and clipping with daubing animal saliva (Figure 2D). We respectively compared the numbers of differential metabolites produced by the two treatments at all three time points and found that 161 differential metabolites due to clipping after 2 h of treatment were recovered after the application of animal saliva. Since this phenomenon was caused by daubing animal saliva, we termed this as the ‘recovery group metabolites’. Meanwhile, 186 differential metabolites were not affected by clipping but newly emerged after the daubing animal saliva treatment. We called the set of metabolites the ‘saliva-specific group metabolites’ (Figure 4A). After 6 h and 24 h treatment, the numbers of differential metabolites in the recovery group were 180 and 138, respectively, and the numbers of differential metabolites in the saliva-specific group were 166 and 214, respectively (Figure 4B,C). It can be seen that the action of animal saliva not only produced unique differential metabolites, but also restored some of the differential metabolites induced by clipping to regular levels. Most of the two types of differential metabolites were distinct at each time point, some were common between the two time points, and a small number of differential metabolites were stably maintained over the three time points (Figure 4D,E). Therefore, the differential metabolites produced due to animal saliva *L. chinensis* not only play roles at a single time point, but also have persistent influence. It suggests the complexity of animal saliva effects.

**Figure 5:**
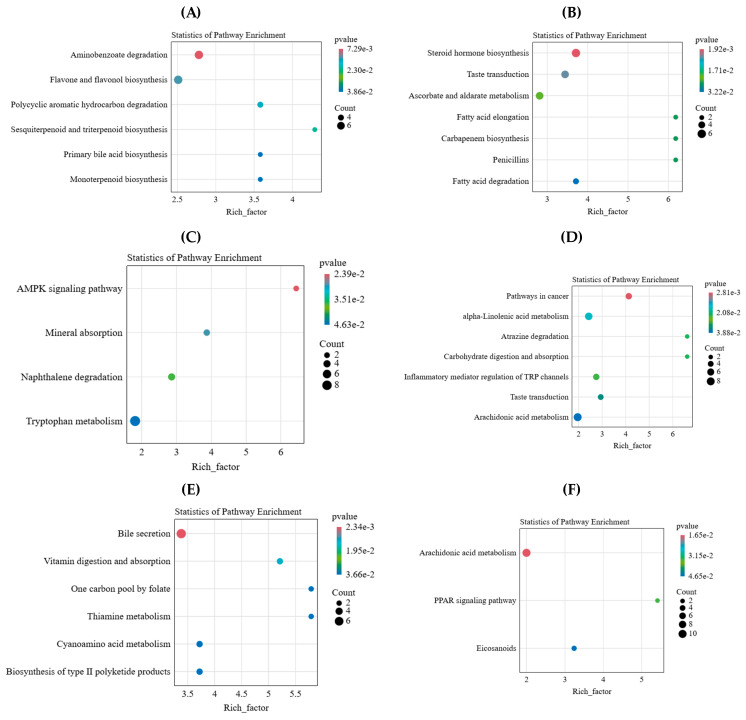
KEGG enrichment analysis of two types of differential metabolites produced by animal saliva action (*p*-value < 0.05 as a significant criterion). (**A**) The recovery group at 2 h after treatment. (**B**) The saliva-specific group at 2 h after treatment. (**C**) The recovery group at 6 h after treatment. (**D**) The saliva-specific group at 6 h after treatment. (**E**) The recovery group at 24 h after treatment. (**F**) The saliva-specific group at 24 h after treatment. The size and color of the bubbles represent the pathway impact and *p*-value of the enrichment analysis, respectively. The darker the color is, the more significant the enrichment.

KEGG pathway enrichment analysis of the two types of differential metabolites showed that the ‘recovery group metabolites’ were enriched to 16 significant metabolic pathways, including aminobenzoate degradation, flavone and flavonol biosynthesis, polycyclic aromatic hydrocarbon degradation, sesquiterpenoid and triterpenoid biosynthesis, primary bile acid biosynthesis, monoterpenoid biosynthesis, the AMPK signaling pathway, mineral absorption, naphthalene degradation, tryptophan metabolism, bile secretion, vitamin digestion and absorption, one carbon pool by folate, thiamine metabolism, cyanoamino acid metabolism, and biosynthesis of type II polyketide products (Figure 5A,C,E). Of note, the metabolic pathways enriched in the saliva-specific group were completely different from those in the recovery group, which included steroid hormone biosynthesis, taste transduction, ascobate and aldarate metabolism, fatty acid elongation, carbapenem biosynthesis, penicillins, fatty acid degradation, pathways in cancer, alpha-Linolenic acid metabolism, atrazine degration, carbohydrate digestion and absorption, inflammatory mediator regulation of TRP channels, arachidonic acid metabolism, the PPAR signaling pathway, and Eicosanoids, the 15 significant metabolic pathways (Figure 5B,D,F). There is a common metabolic pathway, which is taste transduction at the 2 h and 6 h time points, and there is the arachidonic acid metabolism pathway at the 6 h and 24 h time points. Both showed the persistence of animal saliva action.

### 2.4. Key Differential Metabolites and Metabolic Pathways of L. chinensis in Response to Simulated Animal Feeding

**Figure 6:**
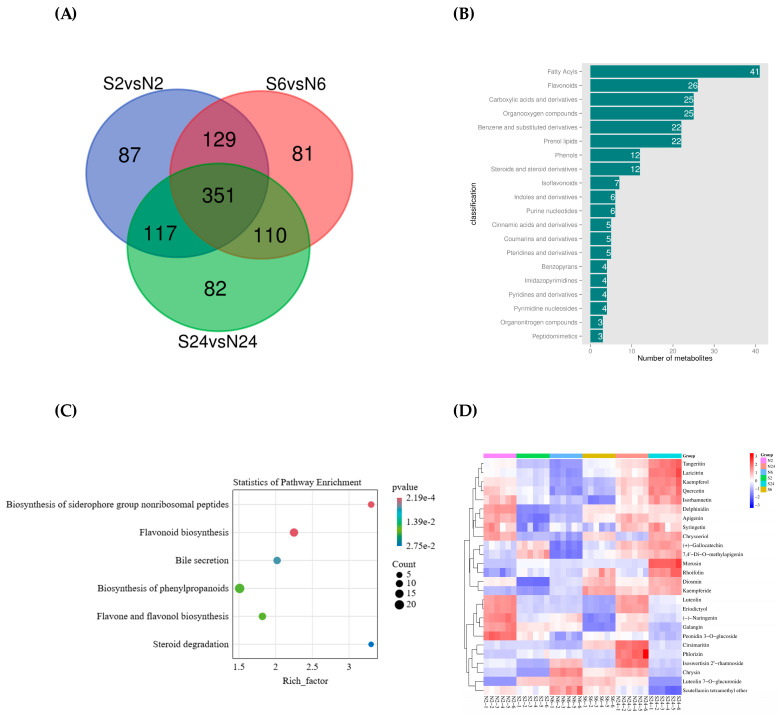
Analysis of key differential metabolites and metabolic pathways produced by animal saliva action. (**A**) Venn diagram showing the common and unique differential metabolites in three different groups. (**B**) Top 20 metabolites of common differential metabolites in HMDB database. (**C**) KEGG enrichment analysis between the common differential metabolites (*p*-value < 0.05 as a significant criterion). (**D**) Clustering heatmap analysis of the flavonoid differential metabolites in common differential metabolites. Red indicates a higher abundance, and blue indicates a lower abundance.

A Venn diagram was drawn by analyzing the presence of differential metabolites at all three time points by comparing the animal saliva treatment group with the clipping treatment group (Figure 6A). The results showed that 351 differential metabolites were shared by the three time points, indicating the changed levels of these 351 metabolites were stable after the initial induction in *L. chinensis*. Thus, these 351 metabolites should be the key differential metabolites in response to animal feeding. According to the HMDB database, these metabolites were classified, and the results showed that the most abundant types were fatty acids (41 in total), followed by flavonoids, which were 26 in total (Figure 6B). The pathways in which the 351 differential metabolites were significantly enriched include the biosynthesis of siderophore group nonribosomal peptides, flavonoid biosynthesis, bile secretion, biosynthesis of phenylpropanoids, flavone and flavonol biosynthesis, and steroid degradation (Figure 6C).

Our results showed that differential metabolites of flavonoids accounted for a relatively large proportion, and the flavonoid biosynthesis pathway was obviously present at all three time points (*p*-value < 0.01), so this metabolic pathway was likely a key metabolic pathway of *L. chinensis* in response to animal feeding. Moreover, 26 kinds of flavonoid differential metabolites showed low expression as a whole at 2 h after clipping and daubing animal saliva treatment. Furthermore, with the extension of time, the number of flavonoid metabolites with high expression also gradually increased; when it was 24 h after daubing animal saliva treatment, there were 13 kinds of flavonoid metabolites with high expression, accounting for half of the 26 kinds of flavonoid compounds (Figure 6D). It indicated that flavonoids metabolites played an active role in the process of responding to the effects of animal saliva, which was consistent with our previous judgment.

### 2.5. The Differentially Expressed Genes (DEGs) Are Enriched in the Flavonoid Biosynthesis Pathway in Response to Simulated Animal Feeding in L. chinensis

In order to further understand the key metabolic mechanism of *L. chinensis* in response to animal feeding, we also explored characteristics of DEGs related to the flavonoid biosynthesis pathway in transcriptomic data of *L. chinensis* in response to animal saliva. Detailed information is provided in the Supplementary Materials (Tables S4–S6). These DEGs can be divided into 11 categories, namely, HCT, FLS, LAR, CHS, CYP73A, ANS, DFR, FNSⅡ, F3′5′H, F3′H, and PGT1 (Table 1). Among them, PGT1 contained the largest number of DEGs. However, each of these three categories (DFR, F3′5′H, and F3′H) had only one gene. Moreover, some DEGs changed at all three time points after the treatment of animal saliva were found in three categories (HCT, FLS, and LAR), indicating saliva effects can be sustained for some genes.

**Figure 7:**
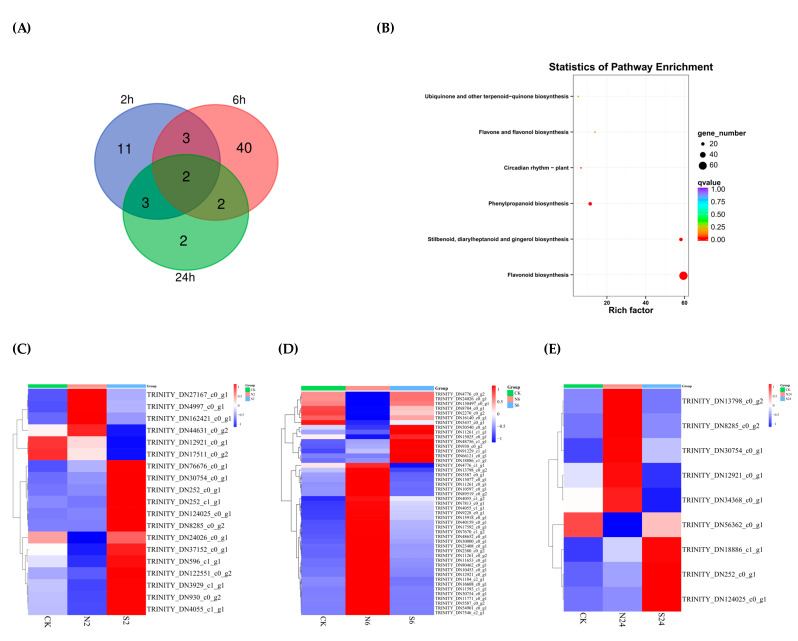
Analysis of differentially expressed genes (DEGs) related to the flavonoid biosynthesis pathway in response to animal saliva in *L. chinensis*. (**A**) Venn diagram showing the numbers of DEGs at three time points. (**B**) KEGG enrichment analysis. (**C**) Clustering heatmap analysis of DEGs at 2 h after treatment. (**D**) Clustering heatmap analysis of DEGs at 6 h after treatment. (**E**) Clustering heatmap analysis of DEGs at 24 h after treatment. Red indicates a higher abundance, and blue indicates a lower abundance.

There were 19, 47, and 9 genes differentially expressed in this pathway after 2 h, 6 h, and 24 h of treatment with daubing animal saliva, respectively. The majority of DEGs existed at only one time point, while a small number of them existed at multiple time points (Figure 7A). TRINITY_DN12921_c0_g1 and TRINITY_DN30754_c0_g1, which belonged to FLS and LAR, were differentially expressed at all three time points after treatment with daubing animal saliva. They may have more important roles in response to animal saliva. Since one gene can regulate multiple metabolic pathways, these DEGs were analyzed for KEGG enrichment, and the results showed flavonoid biosynthesis as the most significant pathway (Figure 7B). The heatmaps showed the expression of them at 2 h, 6 h, and 24 h after treatment (Figure 7C–E). Worthy of note, expression levels of many DEGs converged to control after treatment with daubing animal saliva. These were either highly or lowly expressed during clipping treatment, whereas they showed the opposite expression state after treatment with daubing animal saliva. In short, a significant number of DEGs showed a recovery phenomenon to the control group with daubing animal saliva. This may be the reason why animal saliva can reduce the damage caused by clipping.

## 3. Discussion

In the process of evolution, plants are capable of producing various compounds to cope with changes in the external environment, and flavonoids are an important class of metabolites. As secondary metabolites, flavonoids play an antioxidant role in the plant response to various abiotic stresses. In addition, they are effective endogenous developmental regulators of auxin movement, which plays key roles in controlling the development of individual organs and the whole plants [25,26]. Flavonoids are derived from the phenylpropane metabolic pathway, and their basic structure includes the C15 benzene ring structure of C6-C3-C6. Chalcone synthase (CHS) and Chalcone isomerase (CHI) are key precursor enzymes for flavonoid synthesis [27,28]. The first step in flavonoid biosynthesis is catalyzed by CHS. The substrate converts p-coumaroyl-CoA and malonyl-CoA into naringenin chalcone by CHS, and then naringenin chalcone stereospecific cyclized by CHI. It results in the formation of naringenin, which is the common precursor of flavonols, anthocyanins, proanthocyanidins, flavonoids, and isoflavones. In previous studies, it was found that flavonoids can participate in a variety of stress resistance mechanisms. For example, physiological characterization and transcriptome analysis were used to analyze that flavonoids can promote drought tolerance of maize seedlings by reducing drought-induced oxidative damage and regulating stomatal movement [29]. Excessive accumulation of flavonoids is the key to enhance *Arabidopsis* tolerance to drought stress [30]. The mRNA level of *DoF3H*, a key gene in flavonoid biosynthesis in Dendrobium, was significantly induced by salt stress and low-temperature stress and showed higher tolerance to the stresses in heterogenic *Escherichia coli* [31].

**Figure 8:**
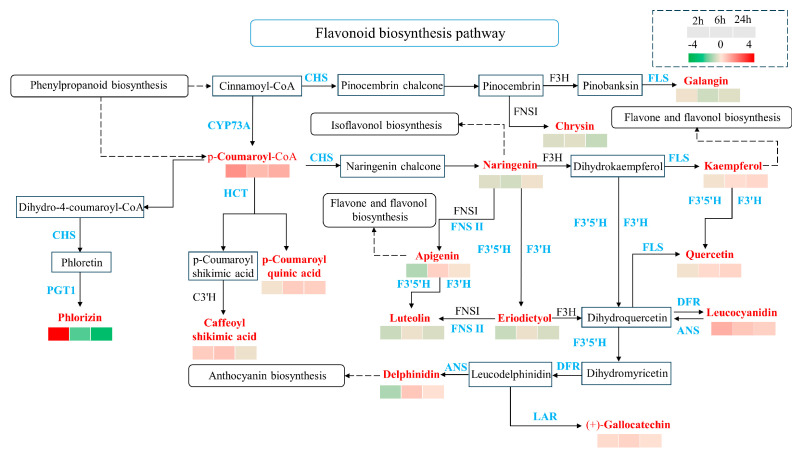
Flavonoid biosynthesis pathway analysis. Differential metabolites are indicated in red, and the heatmap represents the levels of metabolites expression at three times points. Differentially expressed genes are indicated in blue. Solid arrows indicate the relationship of metabolites among the flavonoid biosynthesis pathway. Dotted arrows indicate other metabolic pathways to which the key differential metabolites can be associated.

Our results indicate that flavonoids and the flavonoid biosynthesis pathway played an important role at all three time points (2 h, 6 h, and 24 h) after treatment with daubing animal saliva. By analysis of the flavonoid biosynthesis pathway, a total of 15 differential metabolites showing a variety of expression patterns are identified. Among them, some metabolites maintained the same changed state at the three time points after daubing animal saliva, reflecting a sustained effect of animal saliva. For example, p-Coumaroyl-CoA, Leucocyandian, and (+)-Gallocatechin were continuously up-regulated. While Galangin, Chrysin, Naringenin, Eriodictyol, and Luteolin were down-regulated, some other metabolites showed a regular fluctuation with the extension of time. For example, Kaempferol, Quercetin, p-Coumaryoyl quinic acid, and Delphinidin were down-regulated at 2 h after treatment but up-regulated at 6 h and 24 h. Phlorizin was up-regulated at 2 h, but it was down-regulated at 6 h and 24 h after treatment. In addition, Caffeoyl shikimic acid was up-regulated at 2 h and 6 h after treatment, but it was down-regulated at 24 h. Moreover, Apigenin was up-regulated at 6 h, but it was down-regulated at 2 h and 24 h after treatment (Figure 8). Meanwhile, the DEGs related to the flavonoid biosynthesis pathway also play a critical regulatory role in these differential metabolite changes (Table 1). For example, PGT1 contained 22 down-regulated genes at 6 h after treatment, which may lead to the down-regulation that Phlorizin appeared up-regulated 2 h after treatment and down-regulated 6 h after treatment. In addition, there were four up-regulated genes that appeared in ANS at 6 h after treatment, and one up-regulated gene appeared at 24 h after treatment, which was broadly consistent with the change state of Delphinidin at 6 h and 24 h after the treatment. The remaining 13 differential metabolites are not fully matched with nine kinds of DEGs. According to relevant studies on the evolutionary characteristics of flavonoid metabolism in plants in recent years, this result is not unexpected, that is, the relationship between metabolites and genes is inconsistent [28].

*L. chinensis* produced the recovery phenomenon by clipping with daubing animal saliva treatment at the metabolic level. This recovery phenomenon is related to several metabolic pathways, such as the flavone and flavonol biosynthesis pathway, mineral absorption pathway, etc. Previous studies have shown that the flavone and flavonol biosynthesis and mineral absorption pathway can promote plant growth and yield [32,33]. Therefore, we speculate that animal saliva may promote the growth through these metabolic pathways. It is worth mentioning that in the determination of physiological indexes, animal saliva can reduce the damage caused by clipping. SOD is a key enzyme in the plant defense system. SOD activity decreased under clipping treatment, but increased after clipping with daubing animal saliva. This change may explain why the content of MDA, a harmful peroxide product, increased after clipping but decreased after daubing animal saliva. It can be seen that this recovery phenomenon at the metabolic level is likely to lead to changes in cell-oxidation-related indicators after daubing animal saliva. Moreover, animal saliva can affect the growth through the saliva-specific group. For example, the PPAR signaling pathway has been found to maintain lipid and amino acid metabolism homeostasis during cold stress [34]. Therefore, it can be speculated that animal saliva may maintain the growth of *L. chinensis* by maintaining cellular lipid and amino acid metabolism homeostasis.

In previous studies, flavonoids have been shown to promote the growth of maize seedlings and change the growth state of buckwheat, thus altering its nutritional quality [29,35]. In our study, stable changes in the metabolic pathway of flavonoid biosynthesis in *L. chinensis* induced by animal saliva may promote the regeneration after grazing. In response to the simulated animal feeding process, *L. chinensis* produced many differential metabolites, which were enriched into multiple metabolic pathways, indicating the complexity of the process. According to the relatively large proportion of flavonoid metabolites among 351 distinct and stable differential metabolites found at the three time points after daubing animal saliva treatment, and the apparent existence of the flavonoid biosynthesis pathway at the three time points, our proposition is that the flavonoid biosynthesis metabolic pathway should be a key metabolic pathway in response to simulated animal feeding in *L. chinensis*, and 15 differential metabolites and 11 kinds of DEGs were found in this pathway. Based on the results of this study, it is speculated that the response of *L. chinensis* to the simulated animal feeding process is mainly flavonoid metabolites and the flavonoid biosynthesis pathway, supplemented by a variety of other differential metabolites and metabolic pathways, which jointly promote the complex process of regeneration after grazing. Therefore, the flavonoid biosynthesis pathway may be one of the potential metabolic mechanisms of *L. chinensis* in response to simulated animal feeding.

## 4. Materials and Methods

### 4.1. Leymus chinensis Plants

Within one month after the natural *L. chinensis* returned to green, the well-growing *L. chinensis* seedlings were selected and potted under greenhouse conditions. The mixture of vermiculite and nutritive soil with a ratio of 1:2 was put into a flowerpot with a diameter of 20 cm and a depth of 15.5 cm and filled to a depth of 14 cm. The greenhouse temperature was 25 °C, the humidity was 70%, and 16 h of light (average light intensity 20,000 Lux)/8 h of darkness were set. The same number of seedlings was planted in each pot, and the aboveground part of the *L. chinensis* was cut off, then prepared for experimental treatment until it grew five or six new leaves.

### 4.2. Collection of Animal Saliva

Saliva was collected from the mouths of cows. Before collecting the saliva, the cows were fed with fresh plants. Then, we wrapped a strip of sponge around chopsticks and placed them in the cows’ mouth to keep them chewing on the sponge. We took out the sponge about 2 min later and squeezed the liquid into a cup. The collected saliva was stored between ice bags and processed within two or three hours. All instruments used in the collection of saliva were disinfected with 75% alcohol. During the collection process, the animals did not suffer any physical injury. When cows chewed plants, the collected saliva was consistent with the saliva left on the plant surface during the grazing process.

### 4.3. Treatment of L. chinensis by Simulated Animal Grazing

The *L. chinensis* seedlings from natural grassland to greenhouse were randomly divided into three groups: CK, N, and S. CK was the control, N was the clipped *L. chinensis*, and S was the *L. chinensis* with animal saliva at the cuts. A quarter of the aboveground part of the *L. chinensis* was cut off from the N and S groups for clipping and daubing animal saliva, respectively, while the control group was not cut. All plants in the different time points after processing were sampled respectively, quickly frozen in liquid nitrogen, and stored at −80 °C until processed. The corresponding indexes of the selected plants were determined respectively, and all experiments were performed with at least 3 biological replicates.

### 4.4. Physiological Assays Related to Cellular Oxidation of L. chinensis

Three groups of *L. chinensis* with the same growth were treated, and their leaves at the cut wounds at six hours, the first day, and the third day after clipping were collected. Physiological investigations included two cell oxidation indexes (MDA and SOD), and the specific operation process was carried out in accordance with the instructions from the kit (purchased from Suzhou Keming Biotechnology Co. Ltd. In Suzhou, China).

### 4.5. Widely Untargeted Metabonomic Analysis

Untargeted metabonomic analysis was conducted to analyze the change metabolites of *L. chinensis* in response to clipping and simulated animal feeding at different time points. Metabolite analysis of the *L. chinensis* samples was conducted by Beijing Biomarker Technologies Co., Ltd. (Beijing, China). Metabolic data were processed using the software Analyst 1.6.3. Principal component analysis (PCA) and orthogonal projection latent structure discriminant analysis (OPLS-DA) were used to analyze differences in metabolites between samples. Variable importance projection (VIP, version 1.6.2) of the OPLS-DA model was used to screen for differential metabolites. Metabolites with Fold Change ≥ 1, VIP ≥ 1, and *p*-value < 0.05 were considered differential metabolites. The accumulation of metabolites in *L. chinensis* at various treatments and times was analyzed and compared using the ropls R software package (version 1.6.2) and PCA. The data were normalized, and heatmaps were created to cluster all samples for better visualization.

### 4.6. Transcriptome and Metabolome Analysis of Flavonoid Biosynthesis Pathway in L. chinensis

Based on the analysis of the metabolome data of *L. chinensis*, the flavonoid biosynthesis pathway is found to be that which is closely related to the response to simulated animal feeding processes. Then, differentially expressed genes (DEGs) related to the flavonoid biosynthesis pathway in the *L. chinensis* response to simulated animal saliva at the same time point as metabolome analysis were identified. The DESeq2 R package was used to mine DEGs with the filtering criteria |log_2_FC| ≥ 1 and FDR < 0.01. The fragments per kilobase of transcript per million mapped reads (FPKM) values were calculated and used to evaluate gene expression. KEGG annotation and enrichment analysis of DEGs were obtained using the software KOBAS 3.0. To explore the relationship between the transcriptome and metabolome, a complete map of the flavonoid biosynthesis pathway was summarized by the KEGG pathway database (https://www.genome.jp/kegg/, accessed on 15 January 2024).

## Figures and Tables

**Table 1:** DEGs related to flavonoid biosynthesis pathway in response to animal saliva in *L. chinensis*.

			S2 vs. N2		S6 vs. N6		S24 vs. N24	
Gene Annotation	EC Number	Abbreviation	Up	Down	Up	Down	Up	Down
shikimate O-hydroxycinnamoyl transferase	EC:2.3.1.133	HCT	7	3	4	4	3	1
flavonol synthase	EC:1.14.20.6	FLS		2	1	1		2
leucoanthocyanidin reductase	EC:1.17.1.3	LAR	1			1		1
chalcone synthase	EC:2.3.1.74	CHS	2		2	1		
trans-cinnamate 4-monooxygenase	EC:1.14.14.91	CYP73A	1		2			
anthocyanidin synthase	EC:1.14.20.4	ANS			4		1	
bifunctional dihydroflavonol 4-reductase/flavanone 4-reductase	EC:1.1.1.219/ 1.1.1.234	DFR			1			
flavone synthase II	EC:1.14.19.76	FNSⅡ				1		1
flavanoid 3′,5′-hydroxylase	EC:1.14.14.81	F3′5′H				1		
flavonoid 3′-hydroxylase	EC:1.14.14.82	F3′H				1		
phlorizin synthase	EC:2.4.1.357	PGT1	2	1	1	22		

Note: One unigene may map to more than one enzyme in the pathways. N represents the clipping treated groups, and S represents the animal saliva treated groups. The 2, 6, and 24 represent being treated after 2 h, 6 h, and 24 h, respectively.

## Data Availability

The data used in this study are available from the corresponding author on submission of a reasonable request.

## Supplementary Materials

The following supporting information can be downloaded at: https://www.mdpi.com/article/10.3390/ijms25116110/s1, Table S1: Metabolites annotated in KEGG database; Table S2: Metabolites annotated in HMDB database; Table S3: Metabolites annotated in Lipidmaps database; Table S4: Information of DEGs related to flavonoid biosynthesis pathway in response to animal saliva in *L. chinensis* at 2 h after treatment; Table S5: Information of DEGs related to flavonoid biosynthesis pathway in response to animal saliva in *L. chinensis* at 6 h after treatment; Table S6: Information of DEGs related to flavonoid biosynthesis pathway in response to animal saliva in *L. chinensis* at 24 h after treatment.
